# Integrated Lipidomic and Amino Acid Metabolomic Analyses Reveal Muscle Metabolic Differences in Tibetan Sheep Under Grazing and House-Feeding Systems

**DOI:** 10.3390/ani16132053

**Published:** 2026-07-03

**Authors:** Pengfei Zhao, Jianming Ren, Lan Zhang, Shiyu Tao, Chunyang Li, Ying Ma, Xiong Ma

**Affiliations:** Faculty of Chemistry and Life Sciences, Gansu Minzu Normal University, Hezuo 747000, China

**Keywords:** production systems, meat quality, fatty acids, lipidomics

## Abstract

Production system may affect meat quality and muscle metabolic characteristics in Tibetan sheep. In this study, the biceps femoris muscles of twelve 3-year-old Tibetan sheep with similar body weights were used as experimental materials during a 6-month experimental period. The housed group (n = 6) was defined as the control group (C group), whereas the grazing group (n = 6) was defined as the L group. Meat quality measurement, nutritional composition analysis, untargeted lipidomics, and amino acid metabolomics (AAM) were integrated to investigate the effects of contrasting grazing and house-feeding production systems on meat quality and metabolic characteristics in Tibetan sheep. The results showed that cooking loss and drip loss were significantly decreased, whereas water-holding capacity (WHC) was significantly increased in the L group. However, shear force was also increased, indicating that grazing and house-feeding systems were associated with differences in muscle WHC and shear force. The L group exhibited significant alterations in lipid composition and increased concentrations of several n-3 polyunsaturated fatty acids and increased levels of omega-3 polyunsaturated fatty acids (n-3 PUFAs), including α-linolenic acid (ALA), eicosapentaenoic acid (EPA), and docosahexaenoic acid (DHA), suggesting that grazing and house-feeding systems were associated with differences in the lipid nutritional profile of muscle. Lipidomic analysis showed that the differential lipids were mainly enriched in triacylglycerols (TGs), phosphatidylethanolamines (PEs), and phosphatidylcholines (PCs), and several PUFA-containing TGs and membrane lipid molecules were closely associated with meat quality traits. AAM analysis showed that branched-chain amino acids (BCAAs), including L-leucine and L-valine, as well as N,N-dimethylglycine, were upregulated in the L group, whereas kynurenine and 1-methyl-L-histidine were downregulated. These findings suggest that BCAA metabolism and tryptophan–kynurenine metabolism were associated with metabolic differences observed between production systems in muscle metabolic adaptation. However, amino acid metabolomics analysis revealed that no amino acid metabolites remained significant after FDR correction, and thus the observed pathway-level changes (e.g., BCAA metabolism and tryptophan–kynurenine pathway) should be interpreted as nominal and exploratory findings. Overall, the results indicate that feeding systems were associated with alterations in the lipid and amino acid metabolic profiles of the biceps femoris muscle in Tibetan sheep, which were further associated with differences in muscle WHC, shear force, lipid nutritional composition, and the profile of flavor precursors. This study provides a theoretical basis for optimizing plateau meat sheep production systems and developing high-quality Tibetan sheep meat products.

## Simple Summary

This study investigated the effects of two production systems, indoor feeding and grazing, on the meat quality and nutritional value of Tibetan sheep. The results showed that grazing sheep, likely due to higher activity levels, had muscles with better water-retention capacity and lost less moisture during cooking and storage, although the meat was slightly firmer. The meat from grazing sheep also contained higher levels of beneficial fats, particularly omega-3 fatty acids, and showed changes in compounds related to energy utilization and muscle adaptation. These findings indicate that grazing systems can improve the nutritional composition and water-holding capacity of lamb, while potentially reducing tenderness. This study provides scientific evidence for optimizing Tibetan sheep farming practices and producing healthier, higher-quality lamb.

## 1. Introduction

Compared with plant protein, animal protein generally has higher digestibility and bioavailability, and therefore occupies an important position in dietary protein supply [1]. Tibetan sheep (*Ovis aries*) have become a dominant livestock breed in plateau regions because of their excellent adaptability to high-altitude pastoral environments. Natural grazing has long been the traditional feeding system for Tibetan sheep. However, with the continuous increase in material demand, the scale of Tibetan sheep farming has expanded rapidly, and natural forage resources are no longer sufficient to meet the needs of the current population. Meanwhile, grassland ecosystems are facing increasing pressure. To reconcile the contradiction between the growth of Tibetan sheep numbers and the risk of grassland overgrazing, adjustment of the production system has become the most feasible solution. Currently, Tibetan lamb meat is mainly produced under two production systems: grazing and house-feeding. Compared with grazing systems, house-feeding or intensive finishing systems are generally more conducive to improving growth performance, carcass yield, production efficiency, and the stability of slaughter supply [2,3]. Previous studies have compared meat quality between grazing and house-fed sheep, showing differences in fatty acid composition, intramuscular fat content, shear force, and flavor-related metabolites [4]. However, research on the differences in meat product quality between these two systems remains insufficient, particularly at the molecular levels of lipids and amino acids. As market demand gradually shifts toward high-quality, nutritionally balanced, and functional meat products, systematically evaluating the effects of different production systems on the meat quality and nutritional metabolic characteristics of Tibetan sheep is of great significance for optimizing Tibetan sheep production systems and enhancing the added value of plateau-specific lamb products [3,4,5]. Production systems differ substantially in animal exercise level, and exercise level is one of the important factors affecting the physiological status of skeletal muscle and the formation of meat quality [4,6]. Grazing and house-feeding production systems were associated with differences in shear force, color, water-holding capacity (WHC), flavor compound formation, and nutrient deposition through variations in muscle contractile activity, energy metabolism, fatty acid oxidation, protein turnover, and muscle fiber characteristics. Three-year-old Tibetan sheep were selected because this age represents a physiologically mature stage in which muscle growth has largely stabilized and developmental effects on meat quality are minimized. At this stage, animals have completed major skeletal and muscle development, and metabolic characteristics are relatively stable, thereby reducing confounding effects associated with growth or aging. In addition, this age is commonly encountered in local production systems and is representative of marketable animals under traditional grazing conditions. The biceps femoris (BF) muscle was selected because it is a major hind-limb locomotor muscle and is therefore likely to respond to differences in grazing-associated activity [7]. Therefore, using the BF muscle of Tibetan sheep as the research object to analyze changes in meat quality and metabolic composition under different production systems may help reveal the potential mechanisms by which grazing and house-feeding systems were associated with mutton quality formation.

Intramuscular fat (IMF) is one of the major factors affecting mutton characteristics, including flavor, tenderness, and juiciness [8]. Fatty acids (FAs) are important precursors for the formation of meat aroma and flavor, and their composition can also reflect the nutritional value of meat [9]. Notably, unsaturated fatty acids (UFAs) are more susceptible to oxidative reactions because of the presence of double bonds in their chemical structures [10]. Several polyunsaturated fatty acids (PUFAs), such as C22:6n-3, namely DHA, C20:5n-3, namely eicosapentaenoic acid (EPA), and C20:4n-6, namely arachidonic acid (AA), exert beneficial effects on maintaining body homeostasis and alleviating inflammation. In addition, branched-chain fatty acids (BCFAs), including 4-methyloctanoic acid (MOA), 4-ethyloctanoic acid (EOA), and 4-methylnonanoic acid (MNA), are associated with the formation of a typical “mutton-like odor”, and their levels have been shown to increase with animal age [11]. In addition to lipid composition, amino acids are also important indicators for evaluating the nutritional value and flavor quality of mutton. The content of essential amino acids (EAAs) is directly related to the nutritional value of meat proteins, whereas free amino acids (FAAs) and their derived metabolites are closely associated with the formation of meat taste. Compounds related to umami taste are closely linked to the metabolism of amino acids such as alanine, aspartic acid, and glutamic acid [12]. Therefore, integrating amino acid composition with amino acid metabolite analysis may help explain the formation of meat quality differences in Tibetan sheep under different production systems from nutritional and metabolic perspectives. Mineral elements are also important components of meat nutritional evaluation. Elements such as iron, zinc, calcium, magnesium, and selenium are not only involved in physiological processes, including hematopoiesis, antioxidation, bone development, and regulation of enzyme activity, but they also reflect the nutritional and functional characteristics of meat. Meat is a valuable source of high-bioavailability minerals such as iron and zinc, which contribute significantly to dietary micronutrient intake and are involved in oxygen transport, enzyme function, immune regulation, and other physiological roles [13].

Traditional meat quality indicators can directly reflect phenotypic differences in muscle, but they are insufficient in revealing the underlying metabolic mechanisms through which different production systems affect meat quality formation. To address this, the present study integrates amino acid metabolomics and untargeted lipidomics to systematically dissect, at the molecular level, the intrinsic mechanisms underlying muscle nutrient deposition, flavor precursor formation, and quality differences. We propose the following hypothesis: grazing improves meat quality by remodeling the lipid and amino acid metabolic profiles of the BF in Tibetan sheep. Combining conventional meat quality indicators with multi-omics techniques can establish a comprehensive evaluation framework from “meat quality phenotype” to “nutritional composition” and, in turn, to “metabolic mechanisms”, thereby providing a scientific basis for optimizing Tibetan sheep production systems. Based on this, the present study used the BF muscle of Tibetan sheep subjected to different production systems as the research object. Meat quality traits, amino acid contents, and mineral element composition were systematically determined, and AAM and untargeted lipidomics were further integrated to comprehensively analyze the effects of production systems on muscle quality, nutritional value, and metabolic characteristics in Tibetan sheep. This study aimed to compare differences in meat quality, nutritional composition, and amino acid–lipid metabolic profiles of the BF muscle between house-fed and grazing Tibetan sheep, as well as to explore their potential associations with meat quality traits. The findings provide a theoretical basis for the scientific evaluation of Tibetan sheep meat quality under different production systems, the optimization of plateau meat sheep feeding strategies, and the development of high-quality Tibetan sheep meat products.

## 2. Materials and Methods

### 2.1. Experimental Animals and Sample Collection

Twelve healthy 3-year-old castrated Tibetan sheep, all obtained from Gannan Tibetan Autonomous Prefecture, Gansu Province, China, were selected for this study. The animals were randomly divided into a low-exercise-level group, namely the house-fed group (C group, n = 6), and a high-exercise-level group, namely the grazing group (L group, n = 6). Sheep in the C group were raised in pens with restricted activity space and were fed harvested natural forage, whereas sheep in the L group were managed under natural grazing conditions and traveled daily between the sheepfold and the pasture, covering a round-trip distance of approximately 20 km. The grazing pasture was primarily composed of Poaceae and Cyperaceae species, which are the dominant forage plants in the natural alpine meadow of Gannan. The feeding treatment lasted for 6 months. During the experimental period, all animals had free access to feed and water, and no additional supplementation was provided. Before slaughter, Tibetan sheep were fasted in accordance with animal welfare guidelines. The initial body weights of the house-fed and grazing Tibetan sheep were 40 ± 3.18 kg and 38.25 ± 3.27 kg, respectively, and their average daily weight gains were 79.63 g/day and 55.56 g/day, respectively. After humane slaughter, the BF muscle was immediately separated from the left hind limb, and all visible connective tissues were removed. A portion of the fresh samples was used for meat-quality measurements, including cooking loss and shear force; another portion was snap-frozen in liquid nitrogen for metabolomic analysis; and the remaining samples were used for the determination of nutritional composition and related indicators. It should be noted that the present study was designed to compare the effects of two practical production systems. Therefore, the differences observed between the two groups may reflect the combined effects of feeding system, forage source, grazing activity, and environmental conditions.

### 2.2. Analysis of Technological Meat Quality and Nutritional Value of Skeletal Muscle

The technological meat quality and nutritional composition of muscle samples were determined using standard analytical methods. The measured parameters included the profiles of 37 fatty acids, crude protein content, ash content, and mineral element concentrations, including sodium (Na), potassium (K), calcium (Ca), magnesium (Mg), iron (Fe), manganese (Mn), copper (Cu), zinc (Zn), and phosphorus (P). Meat quality traits, including cooking loss, shear force, drip loss, and WHC, were also evaluated. These measurements were used to comprehensively assess the technological meat quality and nutritional value of meat from different treatment groups.

#### 2.2.1. Determination of Muscle Technological Meat Quality

For the evaluation of muscle technological meat quality, visible fascia was removed, and the muscle samples were trimmed into cuboid pieces of approximately 2 cm × 2 cm × 3 cm. Samples for meat-quality analysis were stored at 4 °C and measured at 24 h postmortem. No additional postmortem aging treatment was applied before analysis. Each sample was weighed using an electronic balance, and the initial weight before cooking was recorded. The samples were then vacuum-sealed in cooking bags and heated in a thermostatic water bath at 85 °C for 20 min to ensure identical heating conditions across all samples. After heating, the samples were cooled to room temperature, gently blotted to remove surface moisture, and weighed again. Cooking loss was calculated as follows: cooking loss (%) = [(initial weight before cooking − final weight after cooking)/initial weight before cooking] × 100, according to previously described methods [14]. Shear force was measured using an MT01 shear force analyzer (Shanghai Boshen Industrial Development Co., Ltd., Shanghai, China). After connective tissue was removed, meat strips of uniform size were prepared along the direction of the muscle fibers, and the samples were sheared perpendicular to the fiber direction. Shear force was determined using a Warner–Bratzler V-shaped blade (Jinan Xiao Electromechanical Co., Ltd., Jinan, China) at a crosshead speed of 250 mm/min. Three measurements were performed for each sample, and the average value was used for statistical analysis. For drip loss determination, deboned muscle samples were weighed and then suspended vertically with steel hooks to maintain a consistent muscle fiber orientation. The samples were placed in inflated self-sealing bags to prevent direct contact between the sample surface and the bag wall, stored at 4 °C for 24 h, and then reweighed for calculation. WHC was determined using the pressing method. Muscle samples without fascia and visible connective tissue were cut into pieces of similar shape along the muscle fiber direction, weighed, and recorded as the initial weight. Each sample was placed between two layers of filter paper and positioned on the testing platform of an RH-1000 meat WHC analyzer (Guangzhou Runhu Instrument Co., Ltd., Guangzhou, China). The sample was pressed under the pressure preset by the instrument. After pressing, the meat sample was removed, surface free water was gently wiped off, and the final weight was recorded. All samples were collected from the same anatomical location, and their weight, shape, and muscle fiber orientation were kept as consistent as possible to improve the comparability of the measurements.

#### 2.2.2. Determination of Meat Nutritional Value

The nutritional value of meat was comprehensively evaluated based on fatty acid composition, crude protein content, IMF content, mineral element concentrations, and ash content. The determination of fatty acid composition was performed with reference to the Chinese National Food Safety Standard GB 5009.168-2016, “Determination of Fatty Acids in Foods” [15]. In brief, homogenized muscle samples were hydrolyzed, lipids were extracted using an ether–petroleum ether mixture, and the extracted lipids were subsequently saponified with sodium hydroxide–methanol solution and methylated with boron trifluoride–methanol solution to generate FAMEs. The derivatized samples were then extracted and diluted with n-hexane, and they were finally analyzed using an Agilent 7890A gas chromatograph equipped (Agilent Technologies (China) Co., Ltd., Beijing, China) with a flame ionization detector (GC–FID). Separation was performed on an HP-88 capillary column (100 m × 0.25 mm i.d., 0.20 μm film thickness). Nitrogen was used as the carrier gas at a flow rate of 10 mL/min. The oven temperature program was set as follows: 100 °C for 15 min, increased to 190 °C at 20 °C/min and held for 6 min, followed by an increase to 220 °C at 1 °C/min and held for 7 min. The injector and detector temperatures were maintained at 260 °C and 250 °C, respectively. A total of 37 fatty acids were quantified using a calibration strategy. Individual fatty acids were identified and quantified by comparison with a 37-component FAME standard mixture, and the fatty acid contents were calculated according to the response factors and conversion coefficients specified in GB 5009.168-2016 [15].

Crude protein content was determined by the Kjeldahl method, which mainly included digestion, distillation, absorption, and titration of muscle samples. The total nitrogen content was calculated according to the volume of acid consumed during titration, and crude protein content was obtained using a nitrogen-to-protein conversion factor of 6.25. IMF content was measured using the classical Soxhlet extraction method. After extraction, the fat collection flask was dried in an oven to constant weight, cooled, and then weighed. Mineral element concentrations were determined by inductively coupled plasma optical emission spectrometry (ICP–OES), tested in accordance with the Chinese National Standard GB 5009.268-2016 [16]. Briefly, single-element standard solutions were diluted with nitric acid to prepare a series of standard solutions with different concentration gradients. Calibration curves were established using PerkinElmer WinLab 32 for ICP software version 5.4, with linear correlation coefficients greater than 0.99. After accurate weighing, muscle samples were transferred into polytetrafluoroethylene digestion vessels, mixed with 5 mL of nitric acid, and subjected to closed-vessel microwave-assisted digestion using a TOPEX microwave digestion system (Shanghai Yiyao Instrument Technology Development Co., Ltd., Shanghai, China). After digestion, the samples were rinsed with ultrapure water and quantitatively transferred into 25 mL volumetric flasks for constant-volume dilution. Elemental identification and quantification were then performed using an ICP–OES instrument (Optima 8000, PerkinElmer, Shelton, CT, USA) according to the established spectral analysis method. Ash content was determined by high-temperature incineration. Ash content was determined according to GB 5009.4-2016 [17]. An appropriate amount of muscle sample was placed in a crucible and first carbonized on an electric furnace at low temperature until no visible smoke was produced. The crucible was then transferred to a muffle furnace and ashed at 550 ± 25 °C for 4 h. The endpoint of ashing was judged based on sample color and the absence of residual carbon particles. After ashing, the crucible was cooled to room temperature in a desiccator and weighed. Blank determination was performed in parallel to ensure the accuracy of the results.

### 2.3. Untargeted Lipidomics Analysis

After homogenization and lipid extraction, muscle samples were subjected to comprehensive UL analysis using an ultrahigh-performance liquid chromatography–Q-Exactive Plus mass spectrometry system, consisting of a Shimadzu Nexera LC-30A UHPLC system (Shimadzu Corporation, Kyoto, Japan) coupled to a Thermo Scientific Q-Exactive Plus mass spectrometer (Thermo Scientific, Waltham, MA, USA). Isotope-labeled internal standards were used for signal correction. Quality control (QC) samples were inserted at regular intervals throughout the analytical sequence to monitor instrumental stability. For lipid extraction, muscle tissue was mixed with 800 μL of methyl-tert-butyl ether (MTBE) and 240 μL of pre-cooled methanol, followed by homogenization. The mixture was then sonicated in a low-temperature water bath for 20 min, equilibrated at room temperature for 30 min, and centrifuged at 14,000× *g* for 15 min at 10 °C. After centrifugation, the upper organic phase was collected, dried under a stream of nitrogen, and reconstituted in 200 μL of 90% isopropanol/acetonitrile for subsequent mass spectrometric analysis. The reconstituted samples were thoroughly vortexed, centrifuged again under the same conditions, and the supernatants were collected for analysis. Chromatographic separation was performed on a C18 column at a column temperature of 45 °C and a flow rate of 300 μL/min. The mobile phases consisted of acetonitrile/water and acetonitrile/isopropanol, and gradient elution was applied. After UHPLC separation, mass spectrometric detection was conducted in both positive and negative electrospray ionization (ESI) modes, with data acquisition performed using the Q-Exactive mass spectrometer (Thermo Scientific, Waltham, MA, USA). The spray voltage was set at 3.0 kV, the source temperature was maintained at 300 °C, the capillary temperature was 350 °C, and the S-Lens RF level was set at 50%. Mass spectra were acquired over an *m*/*z* range of 200–1800. Following each full scan, ten MS/MS spectra were collected using higher-energy collisional dissociation (HCD). The resolutions of MS1 and MS2 were set at 70,000 and 17,500 at *m*/*z* 200, respectively. Lipid annotation was carried out using LipidSearch software version 5.0 (Thermo Scientific, Waltham, MA, USA). The absolute concentrations of target compounds were calculated using the isotope-labeled internal standard method. Lipid species were annotated based on accurate precursor ion masses, MS/MS fragment information, and retention times. For lipid identification, the precursor ion mass tolerance and product ion mass tolerance were both set at 5 ppm, and the product ion threshold was set at 5%. According to the Lipidomics Standards Initiative (LSI) guidelines, lipid species identified based on accurate mass measurements and MS/MS spectral matching, without confirmation using authentic reference standards, were assigned as Level 2 annotations. For lipidomics data processing, lipid features with a detection rate lower than 50% in either group, missing values in more than 50% of samples, or a relative standard deviation (RSD) greater than 30% in QC samples were excluded from subsequent analysis. Lipid features detected in procedural blanks or showing unstable chromatographic peaks were also removed. Missing values were imputed using half of the minimum detected value for each lipid feature. The lipid abundance data were normalized using isotope-labeled internal standards and sample weight, followed by log2 transformation and Pareto scaling before multivariate statistical analysis. QC samples were prepared by pooling equal aliquots from all study samples and were injected at regular intervals throughout the analytical sequence to monitor instrument stability, retention time consistency, and signal reproducibility.

### 2.4. Targeted AAM Analysis

Muscle tissues were thawed at 4 °C, mixed with pre-cooled extraction solvent, homogenized, and sonicated for 30 min. The samples were then incubated at 4 °C for 1 h and centrifuged at 12,000× *g* for 10 min. The supernatant was collected and subjected to solid-phase extraction (SPE), which consisted of four sequential steps: cartridge activation, adsorption of target compounds, impurity elution, and elution of target analytes. The eluate was collected and concentrated to complete dryness, followed by reconstitution in 0.60 mL of 80% methanol/water solution (*v*/*v*). After vortexing for 1 min, the samples were centrifuged at 12,000× *g* for 10 min, and the supernatant was used for liquid chromatography–tandem mass spectrometry (LC–MS/MS) analysis. Chromatographic separation was performed on a reversed-phase column at 35 °C with a flow rate of 0.30 mL/min. The mobile phases consisted of 10 mM ammonium formate aqueous solution as phase A and methanol as phase B. An 8 min gradient elution program was applied, and the injection volume was 6 μL. Mass spectrometric detection was conducted using an ESI source. The curtain gas and collision gas were set to 35 arb and 7 arb, respectively. Peak areas were integrated using MultiQuant software v3.0.3 (SCIEX), and the contents of target compounds were calculated using a one-point internal standard calibration method. Metabolites with poor peak shape, unstable retention time, a missing rate > 50% across samples, or an RSD > 30% in QC samples were removed. Peak areas were normalized using the corresponding internal standards, and missing values were imputed with half of the minimum detected value for each metabolite. Before OPLS-DA and other multivariate analyses, the data were log2-transformed and Pareto-scaled. QC samples were used to assess the reproducibility and stability of the LC–MS/MS analysis. Information on the internal standards used for quantification, the limits of detection (LOD), limits of quantification (LOQ), and QC reproducibility (expressed as relative standard deviation, RSD) for the targeted metabolites are provided in Supplementary Table S1.

### 2.5. Data Processing and Statistical Analysis

All experimental data were organized and tabulated using Microsoft Excel 2013, and statistical analyses were performed with IBM SPSS Statistics 22.0. Continuous variables are presented as mean ± SD. Data normality was assessed using the Shapiro–Wilk test, and homogeneity of variance was evaluated using Levene’s test. For data that satisfied both normality and homogeneity of variance assumptions, differences between the two groups were compared using an independent-samples *t*-test. When the assumption of equal variance was not met, the corrected *t*-test result was used. For lipidomics and targeted AAM analyses, multiple testing correction was performed using the Benjamini–Hochberg false discovery rate (FDR) procedure. Adjusted q-values were calculated based on the original *p*-values. Differential lipids and metabolites were screened using a combination of multivariate and univariate criteria, including VIP ≥ 1 from the OPLS-DA model, *p* < 0.05, and FDR-adjusted q-values where applicable. When metabolites did not remain significant after FDR correction, they were interpreted cautiously as nominally significant exploratory findings. All processed lipidomics and AAM results, including compound names, retention times, ion modes, *m*/*z* values, relative abundances, *p*-values, q-values, and VIP values, are provided in the Supplementary Tables S1–S4. All statistical tests were two-tailed, and differences were considered statistically significant at *p* < 0.05. Although WGCNA generally benefits from larger sample sizes, it has also been applied in exploratory metabolomics and lipidomics studies with limited sample numbers. In the present study, WGCNA was used to identify preliminary lipid co-abundance modules associated with meat quality traits. Therefore, the identified modules and candidate key lipids should be interpreted as exploratory findings requiring further validation in larger independent populations.

## 3. Results

### 3.1. Technological Meat Quality and Nutritional Value of Meat

#### 3.1.1. Technological Meat Quality

Meat quality parameters of the BF muscle, including cooking loss, shear force, drip loss, and WHC, were compared between Tibetan sheep subjected to different production systems. The results showed significant differences in these traits between the two groups. Compared with the C group, the L group exhibited significantly lower cooking loss and drip loss, indicating reduced water loss during storage and heating. Meanwhile, WHC was significantly increased in the L group, further suggesting a stronger water-retention capacity of the muscle. In addition, shear force of the BF muscle was significantly higher in the L group than in the C group, suggesting that grazing and house-feeding systems were associated with differences in muscle structural characteristics and meat tenderness. Overall, the BF muscle from the L group showed better water-retention properties, although meat toughness was relatively increased (Table 1).

#### 3.1.2. Nutritional Composition of Skeletal Muscle

Significant differences in mineral elements and ash content were observed in the BF muscle between the two groups. Compared with the C group, the L group showed markedly higher levels of manganese (Mn), iron (Fe), zinc (Zn), calcium (Ca), and magnesium (Mg), whereas sodium (Na) and ash content were significantly lower. Other mineral elements, including copper (Cu), potassium (K), and phosphorus (P), did not differ significantly between the two groups (Table 2). Overall, these results suggest that grazing and house-feeding systems were associated with differences in the accumulation of specific mineral elements in skeletal muscle, as well as differences in Na deposition and ash content, indicating that grazing and house-feeding systems were associated with variations in mineral deposition and ash composition in skeletal muscle. The analysis of 37 fatty acids, crude protein, mineral elements, and ash content in the BF muscle further revealed significant differences in fatty acid profiles between the two groups (Table 3). Specifically, the L group had significantly higher contents of saturated fatty acids (SFAs), including C15:0, C16:0, C17:0, and C18:0, as well as the monounsaturated fatty acid (MUFA) C18:1n9c, namely oleic acid, than the C group. In contrast, the PUFA C20:4n6, namely AA, was significantly higher in the C group. In addition, the levels of n-3 PUFAs, including C18:3n3, C20:5n3, C22:6n3, α-linolenic acid, EPA, and DHA, were significantly increased in the L group compared with the C group. Consistently, the IMF content was significantly higher in the L group than in the C group, with values of 0.50 ± 0.10% and 0.11 ± 0.06%, respectively. No significant difference in crude protein content was detected between the two groups, suggesting that production systems had a limited effect on protein deposition. Several other fatty acids were not detected because of their low abundance. Taken together, the BF muscle of grazing Tibetan sheep was characterized by increased levels of SFAs, MUFAs, and several n-3 PUFAs, whereas certain PUFAs were relatively higher in the low-exercise group. These findings indicate that production systems can markedly influence meat fatty acid composition, while exerting limited effects on crude protein content.

### 3.2. Lipidomics Analysis

#### 3.2.1. Qualitative and Quantitative Profiling of Total Lipids

**Figure 1:**
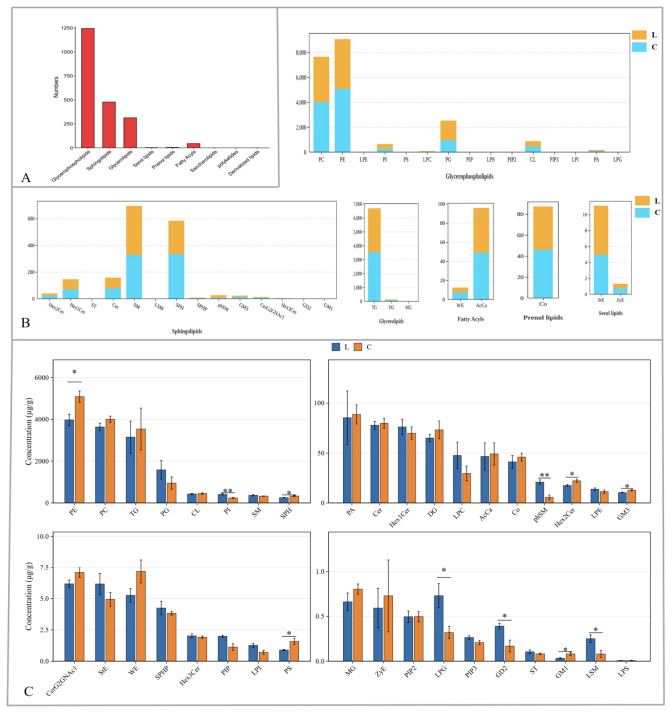
Overview of lipid profiles in the BF of Tibetan sheep. (**A**) Number of identified lipid species in the BF; (**B**) composition of lipid classes; (**C**) concentrations of lipid subclasses. * and ** indicate *p* < 0.05 and *p* < 0.01, respectively.

Lipids in BF muscle were identified in both positive and negative ion modes using the LipidSearch database. A total of 18 samples were analyzed in this study, including six BF samples from the C group, six BF samples from the L group, and six QC samples. In total, 683 lipid species were identified in negative ion mode, whereas 1407 lipid species were detected in positive ion mode. The major lipid categories in muscle tissue were glycerophospholipids, sphingolipids, glycerolipids, and fatty acyls, accounting for 59.57%, 22.87%, 15.02%, and 2.15% of the total identified lipids, respectively (Figure 1A). Further classification showed that glycerophospholipids mainly included PC, PE, phosphatidylinositol (PI), phosphatidylserine (PS), lysophosphatidylcholine (LPC), phosphatidylglycerol (PG), cardiolipin (CL), and phosphatidic acid (PA). Sphingolipids consisted primarily of Hex1Cer, Hex2Cer, ceramide (Cer), sphingomyelin (SM), sphingosine (SPH), and phosphorylated sphingomyelin (phSM). Glycerolipids were mainly composed of TG, diacylglycerol (DG), and monoacylglycerol (MG), whereas fatty acyls mainly included acyl carnitine (AcCa) and wax ester (WE) (Figure 1B).

The lipid metabolites identified in the combined positive and negative ion modes comprised 37 lipid subclasses. Among them, PE (18.47%), PC (17.85%), TG (10.29%), CL (9.76%), Cer (6.94%), Hex1Cer (5.98%), and Hex2Cer (4.74%) were the predominant lipid subclasses (Supplementary Figure S1). The total lipid concentration detected in the L group was 14,337.26 μg/g, whereas that in the C group was 15,450.77 μg/g.

After ranking the lipid subclasses according to their concentrations in the L group, statistical analysis showed that the concentration profiles of lipid subclasses were generally consistent between different production systems. PE, PC, TG, and PG remained among the most abundant lipid subclasses in both groups. Statistical analysis revealed significant differences in several lipid subclasses between the C and L groups. Specifically, PE was significantly higher in the C group than in the L group (*p* < 0.05), whereas PI was significantly higher in the L group than in the C group (*p* < 0.01). In addition, sphingosine (SPH), Hex2Cer, GM3, PS, and GM1 were significantly increased in the C group, while phosphorylated sphingomyelin (phSM), lysophosphatidylglycerol (LPG), GD2, and LSM were significantly increased in the L group (*p* < 0.05 or *p* < 0.01; Figure 1C). Although PC, TG, and PG showed apparent numerical differences between the two groups, these changes did not reach statistical significance and should therefore be interpreted as trends rather than significant alterations. These results suggest that production system may selectively influence specific glycerophospholipid and sphingolipid subclasses in BF muscle.

#### 3.2.2. Correlation Analysis of BF Muscle Lipids Under Different Production Systems

To explore the systematic effects of different production systems on the lipid metabolic network, Pearson correlation coefficients were calculated based on the identified lipid species in each group. Strong lipid–lipid correlations, defined as correlation coefficients greater than 0.8 with *p* < 0.05, were selected to construct lipid correlation networks. The results showed that most lipid species were significantly correlated with each other. A total of 1479 and 1703 strong lipid–lipid correlations were identified in the C and L groups, respectively (Supplementary Table S2). After network construction, key lipids were evaluated according to node degree. In positive ion mode, highly connected nodes were mainly TGs. In negative ion mode, the network hubs in the C group were dominated by phosphatidylinositols (PIs), followed by PEs. By contrast, several PE species in the L group showed enhanced centrality, exceeding that of PIs. Overall, the core lipid nodes in the L group displayed higher connectivity, and the degree values of TGs were markedly increased. The L group also contained more highly connected lipid nodes with degree values greater than 35. In positive ion mode, the degree values of core TGs ranged from 38 to 41 in the L group, which were higher than those in the C group, ranging from 22 to 32 (Supplementary Figure S2). These findings indicate that lipid–lipid correlation patterns were altered in the L group, and that TG and PE species may occupy highly connected positions within the intragroup lipid correlation network.

#### 3.2.3. Analysis of Muscle Lipid Composition

**Figure 2:**
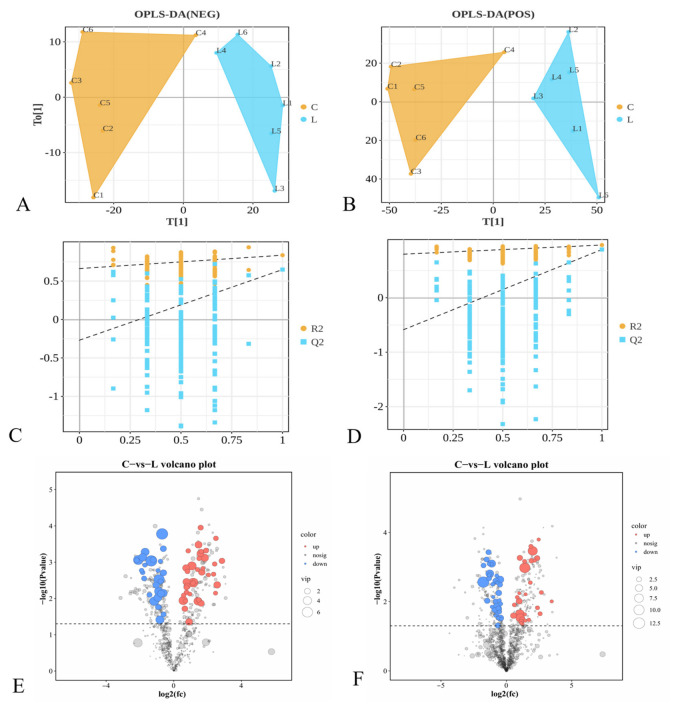
Multivariate analysis and differential lipid screening in positive and negative ion modes. (**A**,**B**): OPLS-DA score plots; (**C**,**D**): Permutation tests; (**E**,**F**): Volcano plots of differential lipids.

Orthogonal partial least squares-discriminant analysis (OPLS-DA) was used to evaluate the lipid profiles of BF muscle between the two groups. Prior to OPLS-DA analysis, an unsupervised principal component analysis (PCA) was performed to obtain an overview of the lipidomic data structure. As shown in Supplementary Figures S1–S5, the quality control (QC) samples were tightly clustered, indicating good analytical stability and reproducibility. Moreover, samples from the C and L groups exhibited a tendency toward separation along the principal component axes, suggesting that the exercise level associated with the production system influenced the overall lipid metabolic profile of BF muscle. The score plots showed a clear separation between the C and L groups in both ion modes (Figure 2A,B), suggesting that the production system markedly affected muscle lipid composition. Cross-validation showed that the R^2^X, R^2^Y, and Q^2^ values were 0.434, 0.846, and 0.696 in positive ion mode, and 0.674, 0.837, and 0.650 in negative ion mode, respectively. The Q^2^ values in both modes were greater than 0.5, and permutation tests showed that the intercepts of the Q^2^ regression lines on the *y*-axis were less than 0 (Figure 2C,D), indicating good predictive performance without obvious overfitting. Lipid species were qualitatively annotated based on MS/MS fragment ion information and database matching, followed by quantitative analysis. Multivariate statistical screening, using variable importance in projection (VIP) ≥ 1 and *p* < 0.05 as the criteria, identified 65 significantly differential lipids in each ion mode. After integrating the results from positive and negative ion modes, 66 lipids were upregulated and 64 lipids were downregulated (Figure 2E,F), indicating that production system exerted a broad regulatory effect on lipid metabolism in BF muscle.

#### 3.2.4. Differential Lipid Profile Analysis of BF Muscle Under Different Production Systems

All significantly differential lipids identified in both ion modes were integrated and quantitatively analyzed. A total of 130 differential lipids were screened between the two exercise-level groups, including 17 CLs, 2 Hex1Cers, 1 Hex2Cer, 2 PAs, 21 PCs, 57 PEs, 6 PGs, 1 phSM, 13 PIs, 1 SM, 3 SPHs, and 6 TGs (Supplementary Figure S3). Among these lipid subclasses, CL, PC, PG, and PE contained both upregulated and downregulated lipid species. In contrast, PI, TG, phSM, and Hex1Cer were only upregulated, whereas PA, SPH, SM, and Hex2Cer were only downregulated (Supplementary Figure S4). Among the upregulated lipids, PE, PG, PI, CL, and TG were the dominant subclasses, comprising 20, 5, 13, 11, and 6 species, respectively. Among the downregulated lipids, PE, PC, CL, and SPH were the major subclasses, comprising 37, 13, 6, and 3 species, respectively. Notably, all differential TGs and PIs were upregulated in the L group. Among all identified lipid species, the most abundant lipids in the C group were PE(39:5e), PC(18:3e_16:0), PE(18:1e_20:4), PE(16:0p_22:4), PC(16:2e_18:0), and PE(18:0_20:4), with concentrations of 555.26, 452.52, 271.34, 247.22, 213.96, and 205.05 μg/g, respectively. In the L group, the most abundant lipids were PE(37:4e), PG(36:0), PG(18:0_19:0), PC(18:3e_16:0), PC(34:3), and PE(16:0p_22:4), with concentrations of 386.05, 375.84, 359.47, 272.78, 196.10, and 177.04 μg/g, respectively. The most prominently upregulated lipids included PC(34:4), PI(18:1_18:2), PC(36:5), and PE(18:1p_18:3), whereas the most strongly downregulated lipids included PE(18:1e_22:3), PE(18:0_20:5), PE(18:0e_22:4), Hex2Cer(d34:6), PE(18:1e_22:4), and PE(39:5e). Notably, all significantly altered TGs were upregulated in the L group, including TG(20:5_18:2_18:2), TG(18:4_16:0_20:5), TG(16:1_18:1_18:3), TG(18:3_18:3_20:4), TG(14:0_18:3_20:5), and TG(18:1_18:2_18:3). These TG species contained PUFA chains, such as C18:2, C18:3, C20:4, and C20:5 (Supplementary Table S3).

It should be noted that although the total annotated lipid level was lower in the L group than in the C group, the differential lipid analysis showed that several PUFA-containing TGs were significantly upregulated in the L group. This suggests that grazing may not simply increase total lipid deposition, but rather remodel the compositional pattern of specific lipid species.

#### 3.2.5. Correlation Analysis of Differential Lipids

**Figure 3:**
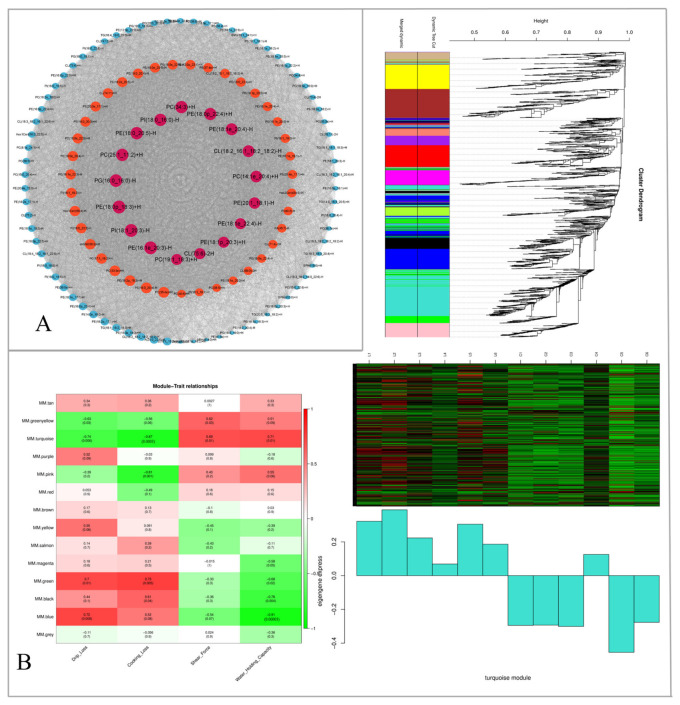
Correlation network and module–trait association analysis of differential lipids. (**A**) Correlation network of differential lipids. Circle size represents betweenness centrality, with larger circles indicating higher centrality values. (**B**) Hierarchical clustering dendrogram of lipid modules and heatmap of module–trait correlations. Red and green indicate positive and negative correlations, respectively, and deeper colors indicate stronger correlations. Values in parentheses indicate *p*-values.

To further investigate the coordinated roles and central positions of differential lipids in the metabolic regulatory network, a correlation network was constructed based on Pearson correlation analysis, in which nodes represented lipid species and edges represented significant correlations with *p* < 0.05. Betweenness centrality was calculated for each lipid node. The results showed that PEs dominated the top 10 central lipids, including PE(16:1e_20:3), PE(18:0p_18:3), PE(18:0_20:5), PE(18:0p_22:4), and PE(18:1e_20:4). The remaining lipids among the top 10 were PI(18:1_20:3), PG(16:0_16:0), PC(25:1_11:2), PI(18:0_16:0), and PC(34:3) (Figure 3A). Receiver operating characteristic (ROC) analysis further indicated that PE(16:1e_20:4), PE(18:0_20:5), and PE(18:1e_20:4) showed potential discriminatory ability between the two groups and could serve as candidate lipid biomarkers. Taken together, these lipids occupied central positions in the overall network and may represent key regulatory molecules. Their perturbation may substantially affect the homeostasis of the lipid metabolic network.

#### 3.2.6. Integrated Analysis of Lipid Modules and Meat Quality Traits

To investigate the associations between lipid modules and meat quality traits of BF muscle, weighted correlation network analysis (WGCNA) was performed to cluster significantly differential lipids into functional modules with highly correlated intra-module abundance patterns. A total of 14 integrated modules were obtained. Module–trait correlation analysis showed that several modules were significantly associated with meat quality traits, among which the turquoise module exhibited the strongest correlations. This module was significantly negatively correlated with cooking loss and drip loss, but significantly positively correlated with shear force and WHC, suggesting that the turquoise module may be closely related to water-retention capacity and shear force changes in BF muscle. By contrast, the blue, green, and black modules were also significantly correlated with certain meat quality traits; however, their associations were more trait-specific and less consistent than those observed for the turquoise module. Therefore, the turquoise module showed stronger associations with multiple meat quality traits in the present samples and may be related to changes in BF muscle quality. Based on intramodular connectivity, module membership, and correlations between metabolites and meat quality traits, PC(34:3)+H, PE(18:1p_20:3)+H, PE(16:1p_20:5)+H, PE(16:2e_20:5)-H, and PI(17:0_20:5)-H were further identified as candidate key lipids. These metabolites were all phospholipid-related molecules and showed consistent correlation patterns with cooking loss, drip loss, shear force, and WHC. These findings suggest that alterations in phospholipid metabolism may represent an important metabolic basis by which production systems affect water-retention capacity and technological meat quality of BF muscle in Tibetan sheep.

### 3.3. Targeted AAM Analysis

**Figure 4:**
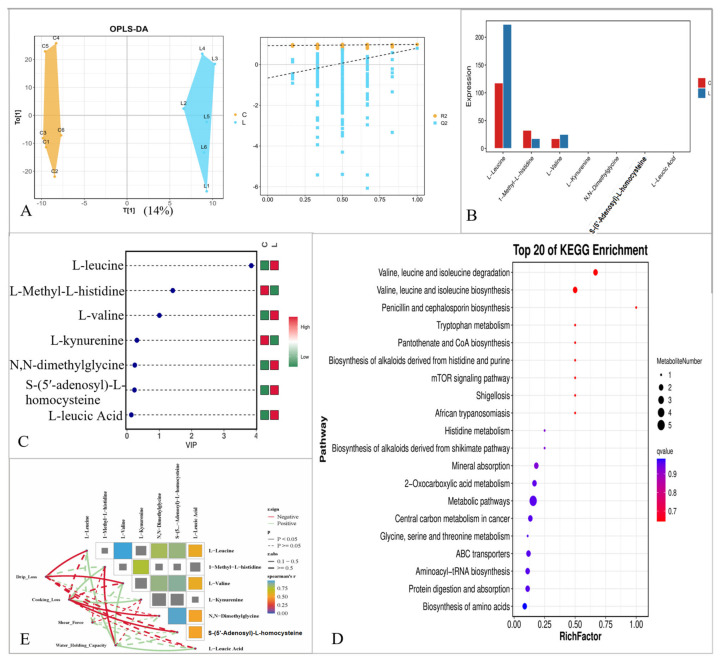
(**A**) OPLS-DA score plot and permutation test results; (**B**) abundance profiles of differential metabolites; (**C**) Variable Importance in Projection (VIP) plot of differential amino acids; (**D**) KEGG enrichment analysis of differential amino acids; (**E**) association analysis between differential amino acids and meat quality traits.

Compound identification was performed based on reference standards by matching Q1/Q3 ion pairs and retention time (RT). Calibration curves were established using authentic standards, and quantitative analysis was conducted with stable isotope-labeled internal standards. Peak areas were normalized to those of the internal standards. OPLS-DA was used to construct a classification model and maximize the separation between the two groups. The score plot showed clear discrimination between the C and L groups. Cross-validation yielded R^2^X = 0.941, R^2^Y = 0.988, and Q^2^ = 0.803, and the permutation test showed a Q^2^ intercept ≤ 0, indicating that the model was robust and had good predictive ability (Figure 4A). An independent-samples Student’s *t*-test, assuming equal variance, was used to screen metabolites differing between the two groups. To account for multiple testing, *p*-values were further adjusted using the Benjamini–Hochberg false discovery rate (FDR) procedure. A total of 36 amino acids and related derivatives were identified in this study, and the complete qualitative and quantitative results are provided in Supplementary Table S4. Seven amino acid-related metabolites showed nominal significance (*p* ≤ 0.05), including five upregulated metabolites in the L group, namely L-leucine, L-valine, N,N-dimethylglycine, S-(5′-adenosyl)-L-homocysteine, and L-leucic acid, and two downregulated metabolites, namely 1-methyl-L-histidine and L-kynurenine. However, none of these metabolites remained statistically significant after FDR correction (q > 0.05). Given the relatively small sample size and the exploratory nature of the targeted AAM analysis, these metabolites were retained for subsequent interpretation based on their nominal statistical significance (*p* ≤ 0.05) and biological relevance. The bar plots showed that L-leucine, 1-methyl-L-histidine, and L-valine were the most abundant metabolites with significant differences between the two groups (Figure 4B). VIP analysis further indicated that these metabolites contributed markedly to the discrimination between the C and L groups (Figure 4C). Kyoto Encyclopedia of Genes and Genomes (KEGG) enrichment analysis showed that the differential metabolites were mainly annotated to BCAA-related metabolic pathways, including valine and leucine metabolism (Figure 4D). These results suggest that BCAA metabolism may participate in BF muscle metabolic remodeling under production systems.

### 3.4. Association Analysis Between Differential Amino Acid-Related Metabolites and Meat Quality Traits

Correlation analysis between differential amino acid-related metabolites and technological meat quality traits showed that L-leucine was strongly positively correlated with L-valine, and S-(5′-adenosyl)-L-homocysteine was also strongly positively correlated with N,N-dimethylglycine. These results indicate that these differential amino acid-related metabolites exhibited coordinated variation patterns across samples. In addition, N,N-dimethylglycine showed strong positive correlations with L-leucine and L-valine, while S-(5′-adenosyl)-L-homocysteine was also positively correlated with L-leucine and L-valine. Regarding technological meat quality traits, L-leucine and L-valine were negatively correlated with drip loss and positively correlated with WHC. N,N-dimethylglycine and S-(5′-adenosyl)-L-homocysteine were negatively correlated with cooking loss, whereas 1-methyl-L-histidine and L-kynurenine were positively correlated with cooking loss. In addition, L-leucine, L-valine, and L-leucic acid showed positive correlation trends with shear force (Figure 4E). Overall, these differential amino acid-related metabolites may participate in the regulation of BF technological meat quality by production system, mainly through their associations with meat water-retention capacity and shear force-related traits.

## 4. Discussion

Meat quality is an important indicator for evaluating the eating experience and nutritional value of meat, and is jointly influenced by multiple factors, including breed, age, sex, muscle type, postmortem handling, and feeding management practices [18]. The main phenotypic finding of this study was that the L group exhibited better water-retention capacity but higher shear force, suggesting that the grazing production system may be associated with improved moisture retention but increased mechanical strength of meat. Therefore, the effect of grazing and house-feeding systems on meat quality was not simply beneficial in one direction, but rather reflected a trade-off among water retention, shear force, and structural properties. This finding is generally consistent with previous understanding of the factors affecting mutton quality, whereby exercise, feeding management, muscle fiber characteristics, and energy metabolism may jointly contribute to variations in meat quality [19]. Previous studies have also shown that long-term exercise can induce changes in muscle fiber phenotype and non-coding RNA (ncRNA) regulatory networks, which are associated with IMF deposition and improved meat quality, The study showed that the intramuscular fat (IMF) of the grazing group was significantly higher than that of the confined group, and previous studies used the same samples as those in this study [20]. However, it should be noted that the increase in IMF did not lead to a reduction in shear force in the L group, indicating that IMF is not the sole determinant of tenderness. Shear force may also be influenced by meat fiber structure, connective tissue content, the degree of collagen cross-linking, and postmortem aging. Therefore, the concurrent increase in IMF and shear force observed in this study is not contradictory, but may reflect the dual regulatory effects of grazing-associated exercise on lipid deposition and the mechanical structure of meat.

The present findings are not entirely consistent with some studies, suggesting that house feeding may improve meat quality in Tibetan sheep. Previous research has reported that house-fed Tibetan sheep may show advantages over grazing Tibetan sheep in terms of fat deposition, shear force, and certain flavor-related metabolites [4]. The discrepancy may be partly explained by differences in the nutritional level of “house feeding” across studies. In some previous studies, house-fed animals were usually provided with nutritionally balanced finishing diets, whereas the C group in the present study was closer to a traditional roughage-based feeding system. Previous evidence has also indicated that oxidative muscle fibers are closely associated with lipid metabolism and IMF characteristics [21]. Therefore, the C group in this study should not be simply equated with intensive finishing systems used in other studies, which may partly account for the inconsistent findings. Grazing-associated factors, including exercise and forage intake, may promote oxidative metabolic adaptation in muscle and consequently enhance the deposition and turnover of specific lipid species; nevertheless, the overall outcome remains dependent on the balance between energy intake and energy expenditure.

The fatty acid profile further indicated that grazing and house-feeding systems were associated with differences in the nutritional lipid characteristics of Tibetan sheep meat. This observation is consistent with recent livestock metabolomic and lipidomic studies, showing that feeding regime, muscle type, and intramuscular lipid remodeling can markedly influence fatty acid composition, flavor precursor formation, and meat quality traits [4,22]. In the present study, the L group showed increased levels of n-3 PUFAs, including α-linolenic acid, EPA, and DHA, whereas certain n-6 fatty acids, such as AA, were reduced. These differences suggest that the grazing and house-feeding systems were associated with distinct fatty acid compositions in BF muscle, particularly with respect to the n-6/n-3 fatty acid profile. The observed enrichment of n-3 polyunsaturated fatty acids (PUFAs) in the L group may be closely related to differences in forage resources associated with the grazing production system. Fresh pasture is generally rich in α-linolenic acid, which serves as the primary dietary precursor of long-chain n-3 PUFAs in ruminants. Although a substantial proportion of dietary ALA undergoes ruminal biohydrogenation, part of it can escape this process and be absorbed and deposited in muscle tissues. In addition, ALA can be further converted through elongation and desaturation pathways to produce longer-chain n-3 fatty acids, including EPA and DHA. Therefore, the higher concentrations of ALA, EPA, and DHA observed in the L group may partly reflect the greater intake of pasture-derived n-3 fatty acids and their subsequent metabolic conversion and deposition in muscle. Fatty acids are not only nutritional components but also important precursors of volatile flavor compounds generated during thermal processing of meat [23]. However, higher levels of UFAs may contribute to the formation of more lipid oxidation-derived flavor precursors, while excessive oxidation may also reduce flavor stability. Therefore, the present results only indicate that the grazing and house-feeding systems were associated with differences in fatty acid composition related to nutritional value and flavor precursor formation. However, whether these differences ultimately influence sensory flavor characteristics requires further validation.

In addition, mineral element analysis showed that the contents of Mn, Fe, Zn, Ca, and Mg in the BF muscle were significantly higher in the L group than in the C group, whereas Na and ash content were significantly decreased. These results suggest that grazing and house-feeding may affect mineral deposition in muscle. Fe, Zn, Ca, and Mg are important elements for evaluating the nutritional value of meat and are closely associated with physiological processes such as heme protein formation, enzyme activity regulation, muscle contraction, and energy metabolism [24,25]. Therefore, the increased levels of certain mineral elements in the L group may be associated with higher activity level, oxidative metabolic adaptation in muscle, and enhanced mineral deposition. However, muscle mineral composition is also influenced by forage type, pasture mineral status, and feeding behavior [26].

Lipidomic results showed that the L group did not simply exhibit a general increase in total lipid abundance; instead, specific lipid species and the lipid network structure were remodeled. Similar lipidomic evidence from livestock muscle studies has indicated that TG, PC, PE, and related phospholipid species are closely associated with intramuscular fat deposition, oxidative stability, flavor development, and muscle-specific quality variation, suggesting that lipid remodeling may represent a conserved metabolic feature underlying meat quality formation [22,27]. The differential lipids were mainly distributed among TG, PE, PC, PI, and CL subclasses. In particular, several PUFA-containing TGs were upregulated in the L group, suggesting that the grazing and house-feeding systems were associated with differences in the accumulation of lipid species related to energy storage. Meanwhile, membrane lipid species such as PE, PC, and PI showed high connectivity in the correlation network, suggesting that membrane lipid metabolism may be associated with variations in muscle cell membrane characteristics, mitochondrial function, and water-retention capacity. Previous studies have demonstrated that phospholipids, including PC, PE, CL, and PI, are important components of meat lipids and are closely related to oxidative stability and flavor formation in meat [27]. Previous multi-omics evidence further suggests that lipid species, including TG, PC, and PE, contribute to flavor variation among different mutton muscle cuts [22]. Therefore, the lipid network remodeling observed in this study may represent a metabolic feature associated with differences between grazing and house-feeding systems and may represent candidate metabolic pathways associated with differences between production systems. AAM results showed that BCAAs, such as L-leucine and L-valine, were upregulated in the L group, whereas L-kynurenine was downregulated. BCAAs are not only important components of muscle proteins, but are also involved in muscle protein turnover, energy metabolism, and mTOR-related signaling regulation [28]. In this study, BCAAs were correlated with meat quality traits such as drip loss, WHC, and shear force, suggesting that they may be associated with muscle quality variation between grazing and house-feeding systems. BCAAs, such as L-leucine and L-valine, are key regulators of muscle protein turnover and energy metabolism through pathways such as mTOR/p70S6K signaling [29,30]. Moreover, kynurenine metabolism is closely associated with inflammation, oxidative stress, and stress-related metabolic regulation, and exercise-induced skeletal muscle adaptation has been reported to modulate kynurenine metabolism and stress resilience [31]. However, increased BCAA levels cannot be simply equated with improved flavor, because amino acids such as L-leucine and L-valine may also have bitter-taste characteristics. Their final contribution to flavor should therefore be evaluated together with umami-related amino acids, nucleotides, and volatile flavor compounds [32]. In addition, L-kynurenine, an important intermediate in tryptophan metabolism, is closely associated with inflammation, oxidative stress, and muscle metabolic status. The lower level of L-kynurenine observed in the L group may reflect differences in metabolic status associated with the grazing and house-feeding systems [33].

A limitation of this study is that only 3-year-old sheep were included. Therefore, the results may not fully represent younger or older age groups. Future studies should consider multiple age categories to further explore the interaction between age and production system on meat quality. Several limitations of this study should be acknowledged. First, although significant differences in meat quality, lipid metabolism, amino acid metabolism, and mineral composition were observed between the two production systems, the effects of grazing-associated exercise could not be completely separated from those of forage source, pasture composition, and feeding behavior. The chemical composition of natural pasture and harvested forage, as well as individual feed intake, were not systematically quantified. Therefore, the observed metabolic and meat quality differences likely reflect the combined effects of exercise level and dietary factors. Second, the sample size was relatively small (n = 6 per group), which may limit statistical power and the robustness of correlation analyses involving lipidomic and amino acid metabolomic data. Consequently, the identified metabolic biomarkers and their associations with meat quality traits should be interpreted cautiously and validated in larger populations. Third, although the grazing group exhibited higher intramuscular fat (IMF) content, WHC, and shear force, these changes could not be fully interpreted due to the lack of direct structural and biochemical measurements. Muscle histological characteristics, such as fiber-type composition and diameter, connective tissue distribution, collagen content and cross-linking, as well as postmortem muscle pH, were not evaluated. These factors are known to influence tenderness and meat texture, and their absence limits mechanistic interpretation of why IMF and shear force increased concurrently. Therefore, the observed increase in both IMF and shear force likely reflects a combination of enhanced lipid deposition and structural adaptations of muscle under grazing conditions, but the precise mechanisms remain speculative. Future studies integrating muscle histomorphology, collagen characteristics, postmortem pH, forage composition, and multi-omics analyses in larger animal populations will help clarify the mechanisms through which grazing production systems influence muscle structure, meat quality, and metabolic remodeling in Tibetan sheep.

## 5. Conclusions

Grazing and house-feeding production systems were associated with significant differences in meat quality traits, lipidomic composition, and amino acid metabolic profiles of BF muscle. Compared with the C group, the L group showed lower cooking loss and drip loss, together with higher WHC, IMF content, n-3 PUFA levels, and shear force. These results suggest that the grazing system may confer advantages in water-retention capacity and nutritional lipid composition, while also increasing muscle structural strength and potentially affecting tenderness. At the nutritional and metabolic levels, the L group exhibited increased IMF content and higher levels of n-3 PUFAs, including α-linolenic acid, EPA, and DHA. Lipid species such as TG, PE, PC, and PI, as well as BCAAs represented by L-leucine and L-valine, were significantly altered and were correlated with meat quality traits. Overall, the differences observed between the grazing and house-feeding systems were associated with variation in water-retention capacity, shear force, nutritional lipid composition, and metabolic profiles of BF muscle in Tibetan sheep. These findings provide preliminary evidence that grazing and house-feeding systems are associated with differences in meat quality traits and metabolic characteristics of Tibetan sheep muscle. The results also provide a theoretical basis for further understanding the relationships between production systems, muscle metabolism, and meat quality, and they may contribute to the optimization of plateau sheep production systems and the development of high-quality Tibetan sheep meat products.

## Figures and Tables

**Table 1:** Comparison of technological meat quality traits of skeletal muscle between different production systems.

Metrics	Groups		*p*-Value
	C	L	
Cooking loss (%)	30.62 ± 2.19 ^a^	27.27 ± 2.42 ^b^	*p* < 0.05
Shear force (N)	59.84 ± 2.53 ^B^	64.26 ± 1.88 ^A^	*p* < 0.01
Drip loss (%)	7.13 ± 0.57 ^A^	5.92 ± 0.84 ^B^	*p* < 0.01
Water-holding capacity (%)	61.00 ± 2.31 ^b^	64.08 ± 1.93 ^a^	*p* < 0.05

Different superscript letters indicate significant differences (A, B: *p* < 0.01; a, b: *p* < 0.05). The same applies below.

**Table 2:** Comparison of mineral composition in BF muscle between groups.

Parameters	Mean ± SD		*p*-Value
	C	L	
Mn (mg/kg)	0.112 ± 0.018 ^B^	0.198 ± 0.011 ^A^	*p* < 0.001
Fe (mg/kg)	12.418 ± 0.827 ^B^	20.400 ± 1.122 ^A^	*p* < 0.001
Cu (mg/kg)	0.442 ± 0.025	0.483 ± 0.028	*p* = 0.310667
Zn (mg/kg)	20.700 ± 1.130 ^b^	23.900 ± 0.679 ^a^	*p* < 0.05
K (×10^3^) (mg/kg)	4.447 ± 0.051	4.340 ± 0.082	*p* = 0.30
Ca (mg/kg)	32.483 ± 1.187 ^B^	43.950 ± 1.347 ^A^	*p* < 0.001
Na (mg/kg)	554.667 ± 21.403 ^A^	423.000 ± 14.731 ^B^	*p* < 0.001
Mg (mg/kg)	200.333 ± 5.560 ^b^	219.333 ± 4.055 ^a^	*p* < 0.05
P (×10^3^) (mg/kg)	3.662 ± 0.102	3.635 ± 0.037	*p* = 0.813048
Ash content (g/100 g)	1.600 ± 0.051 ^A^	1.083 ± 0.040 ^B^	*p* < 0.001

Different superscript letters indicate significant differences (A, B: *p* < 0.01; a, b: *p* < 0.05). The same applies below.

**Table 3:** Comparison of FAs and CP in skeletal muscle under different production systems.

Meat Composition	Mean ± SD (g/100 g)		*p*-Value
	C	L	
C14:0	0.023 ± 0.005	0.023 ± 0.003	*p* = 0.97
C15:0	0.003 ± 0.000 ^b^	0.005 ± 0.001 ^a^	*p* < 0.05
C16:0	0.219 ± 0.016 ^b^	0.337 ± 0.034 ^a^	*p* < 0.05
C16:1	0.014 ± 0.002	0.018 ± 0.003	*p* = 0.271
C17:0	0.010 ± 0.001 ^b^	0.016 ± 0.002 ^a^	*p* < 0.05
C18:0	0.207 ± 0.007 ^b^	0.322 ± 0.044 ^a^	*p* < 0.05
C18:1n9c	0.326 ± 0.027 ^b^	0.595 ± 0.076 ^a^	*p* < 0.05
C18:2n6c	0.075 ± 0.006	0.070 ± 0.005	*p* = 0.481
C18:3n3	0.002 ± 0.000 ^B^	0.027 ± 0.002 ^A^	*p* < 0.001
C20:4n6	0.044 ± 0.003 ^A^	0.026 ± 0.002 ^B^	*p* < 0.01
C20:5n3	0.002 ± 0.000 ^B^	0.012 ± 0.000 ^A^	*p* < 0.001
C22:6n3	0.003 ± 0.000 ^B^	0.006 ± 0.001 ^A^	*p* < 0.01
CP	20.450 ± 0.628	20.483 ± 0.421	*p* = 0.97

Different superscript letters indicate significant differences (A, B: *p* < 0.01; a, b: *p* < 0.05). The same applies below.

## Data Availability

The original contributions presented in this study are included in the article/Supplementary Materials. Further inquiries can be directed to the corresponding author.

## Supplementary Materials

The following supporting information can be downloaded at: https://www.mdpi.com/article/10.3390/ani16132053/s1, Figure S1: Proportion distribution of lipid metabolite sub-subclasses identified under combined positive and negative ionization modes; Figure S2: Intragroup correlation analysis of lipids in Group C and Group L; Figure S3: Subclasses and relative abundance of differentially abundant lipids; Figure S4: The number of up-regulated and down-regulated lipids; Figure S5: PCA score plot of lipidomic profiles including QC samples; Table S1: Internal standards, LOD, LOQ, and QC reproducibility (RSD) of targeted metabolites; Table S2: lipid–lipid correlation information; Table S3: Significantly up- and down-regulated lipid species between the C and L groups; Table S4: Complete qualitative and quantitative results of 36 amino acids and related derivatives.
