# Supplementary material for: Integrated Lipidomic and Amino Acid Metabolomic Analyses Reveal Muscle Metabolic Differences in Tibetan Sheep Under Grazing and House-Feeding Systems
Source: Animals (Basel). 2026 Jul 3;16(13):2053. doi: 10.3390/ani16132053 (PMC13360594; doi:10.3390/ani16132053)
Supplement: Supplementary file 1 [file animals-16-02053-s001.zip › Supplementary Figure.pdf]

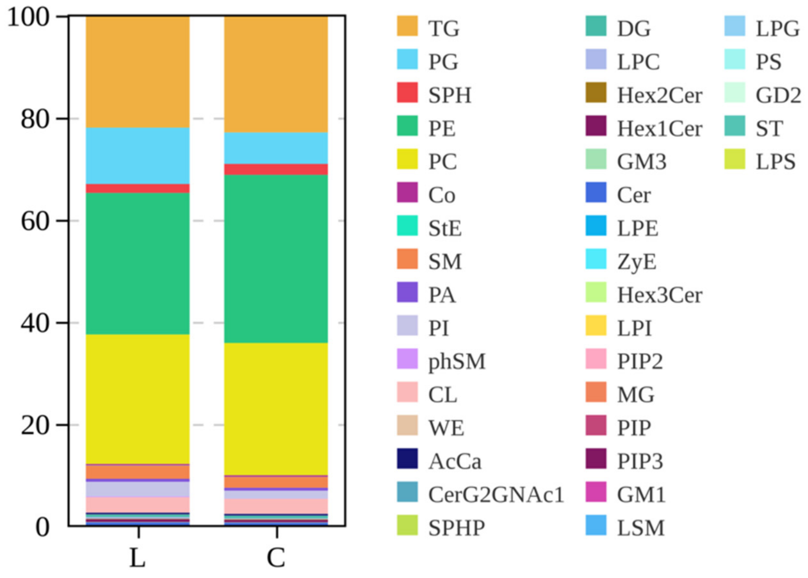

Supplementary Figure S1. Proportion distribution of lipid metabolite sub-subclasses identified under combined positive and negative ionization modes.

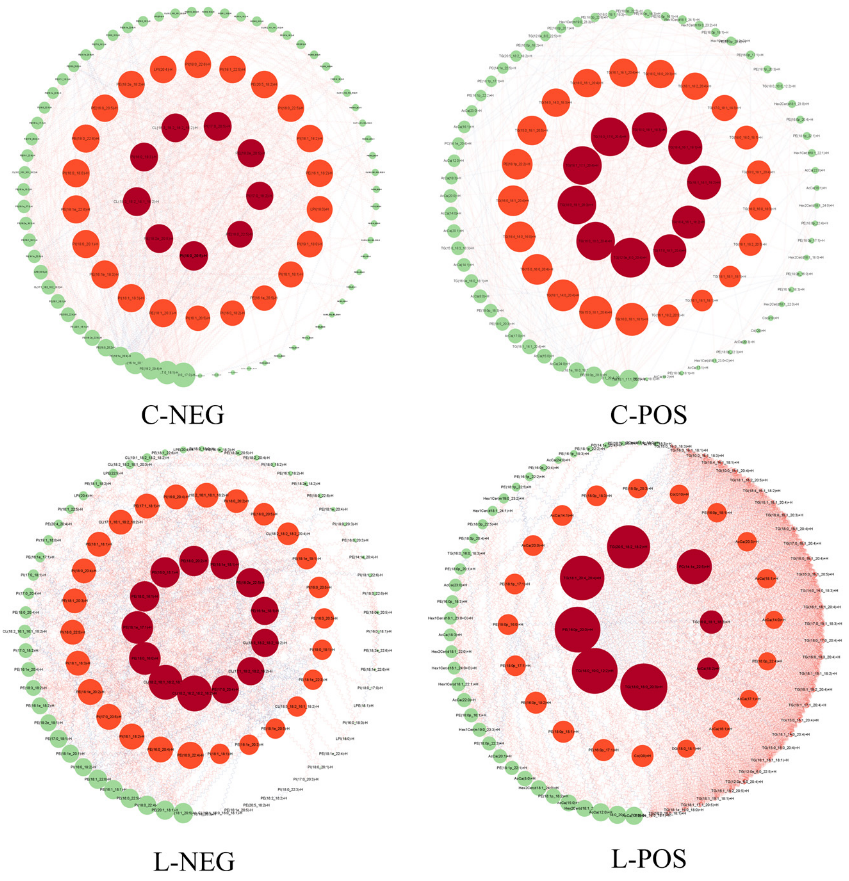

Supplementary Figure S2. Intragroup correlation analysis of lipids in Group C and Group L.

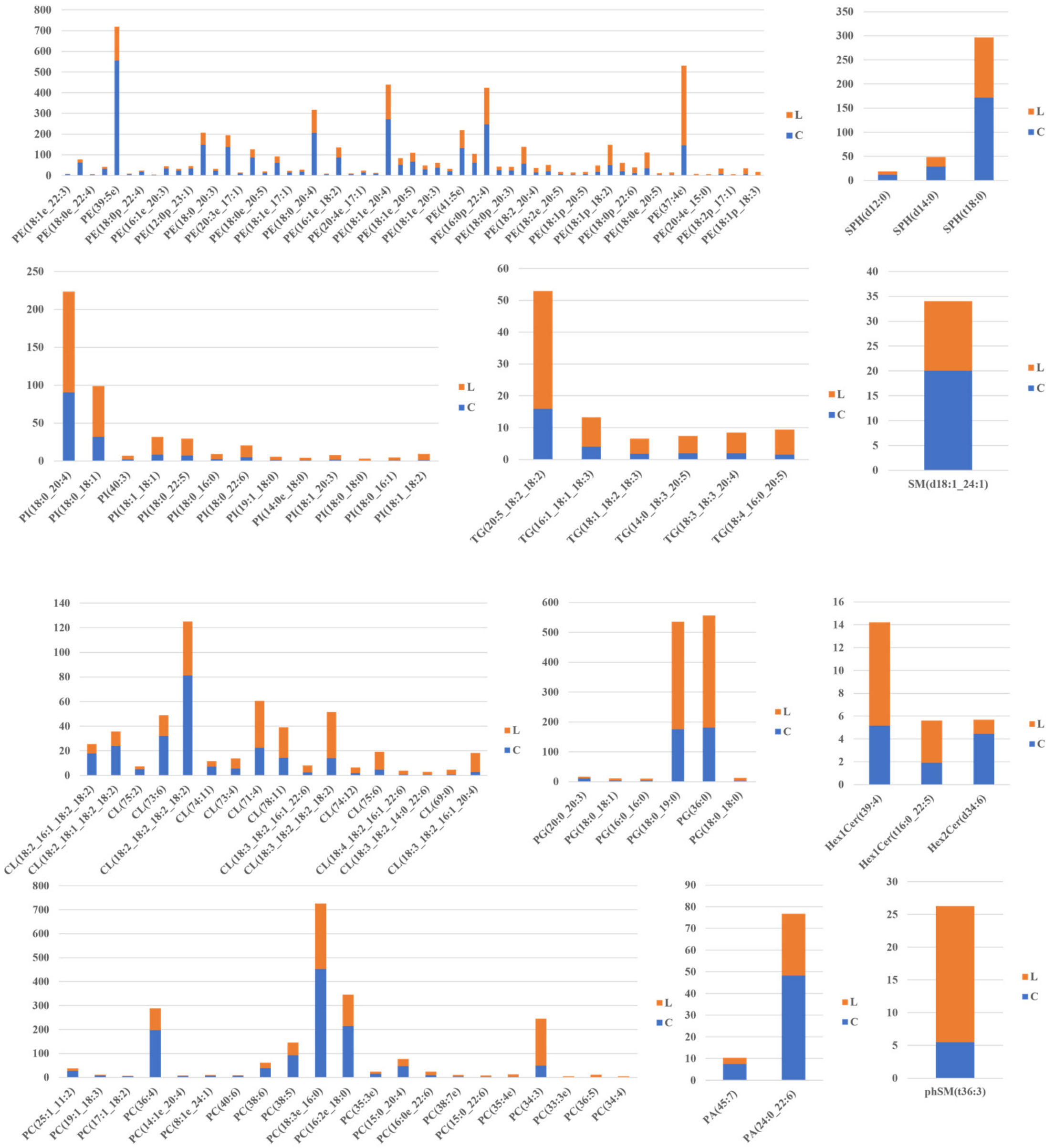

Supplementary Figure S3. Subclasses and relative abundance of differentially abundant lipids.

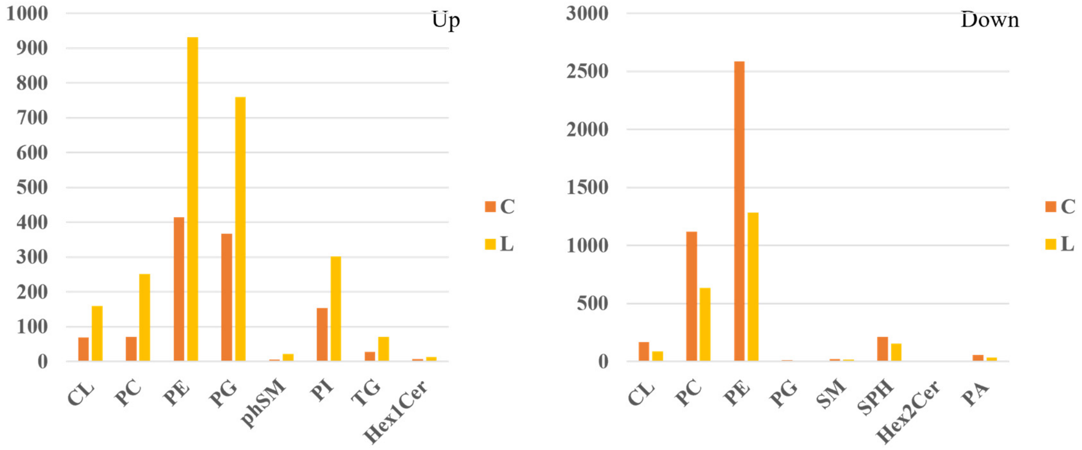

Supplementary Figure S4. The number of up-regulated and down-regulated lipids.

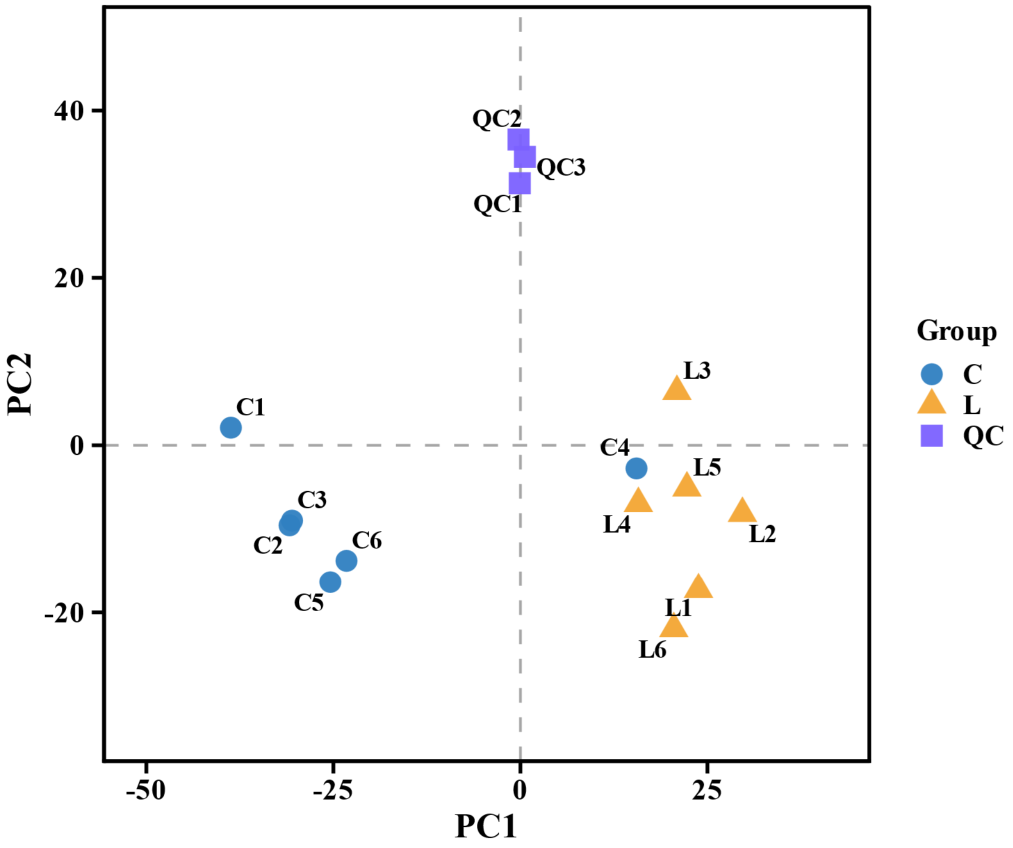

Supplementary Figure S5. PCA score plot of lipidomic profiles including QC samples
